# Affect-Driven Attention Biases as Animal Welfare Indicators: Review and Methods

**DOI:** 10.3390/ani8080136

**Published:** 2018-08-07

**Authors:** Andrew Crump, Gareth Arnott, Emily J. Bethell

**Affiliations:** 1 Institute for Global Food Security, School of Biological Sciences, Queen’s University Belfast, Medical Biology Centre, 97 Lisburn Road, Belfast BT9 7BL, UK; g.arnott@qub.ac.uk; 2 Research Centre in Brain and Behaviour, School of Natural Sciences and Psychology, Liverpool John Moores University, James Parsons Building, Byrom Street, Liverpool L3 3AF, UK; E.J.Bethell@ljmu.ac.uk

**Keywords:** animal welfare, cognitive bias, attention bias, looking time, emotional Stroop, dot-probe, spatial cueing, visual search, broaden-and-build theory, attention bias modification

## Abstract

**Simple Summary:**

Good animal welfare requires minimizing suffering and promoting positive experiences. To achieve this, we need reliable indicators of animals’ psychological states. In humans, different moods and emotions (“affects”) are associated with changes in visual attention (“attention bias”). We review studies investigating whether attention biases are also indicators of affect in animals. Although research is limited, evidence for affect-driven attention biases has been found in several species, especially primates and livestock. These studies are discussed in relation to tasks developed for measuring attention in humans. We identify additional findings from human psychology that might be applied to animals, particularly species not studied before, and conclude that affect-driven attention bias is a promising welfare indicator. However, it may be more useful for studying responses to specific stimuli, rather than general wellbeing. With further study, we hope these findings contribute to fulfilling society’s ethical obligations towards animals.

**Abstract:**

Attention bias describes the differential allocation of attention towards one stimulus compared to others. In humans, this bias can be mediated by the observer’s affective state and is implicated in the onset and maintenance of affective disorders such as anxiety. Affect-driven attention biases (ADABs) have also been identified in a few other species. Here, we review the literature on ADABs in animals and discuss their utility as welfare indicators. Despite a limited research effort, several studies have found that negative affective states modulate attention to negative (i.e., threatening) cues. ADABs influenced by positive-valence states have also been documented in animals. We discuss methods for measuring ADAB and conclude that looking time, dot-probe, and emotional spatial cueing paradigms are particularly promising. Research is needed to test them with a wider range of species, investigate attentional scope as an indicator of affect, and explore the possible causative role of attention biases in determining animal wellbeing. Finally, we argue that ADABs might not be best-utilized as indicators of general valence, but instead to reveal specific emotions, motivations, aversions, and preferences. Paying attention to the human literature could facilitate these advances.

## 1. Introduction

Animal welfare science aims to reduce suffering and improve wellbeing [1,2]. To achieve this, we need objective measures and standardised tests that quantify the psychological component of affect. However, animals cannot communicate verbally and we cannot measure their subjective experience directly [3]. Welfare scientists instead rely on indirect indicators, such as behaviour and physiology. This can be problematic, though, because behaviour is often species-specific, difficult to interpret, and varies between individuals (personality). It may only highlight extremes of welfare and can become dissociated from affective state, as in stereotypies [4]. Physiology, meanwhile, fluctuates with activity level and circadian rhythms, often signalling affective intensity (arousal) rather than valence (see Reference [5]). Moreover, behavioural and physiological welfare indicators have traditionally focused on negative affect, but good welfare also requires recognizing and promoting positive states [2]. Additional measures are therefore needed.

A promising avenue of research for measuring affective states in animals comes from cognitive psychology (see Reference [6]). In humans, theory and methods to investigate the relationship between affect, cognition, and subjectively experienced feelings are well established. For example, people in negative-valence states recall more negative memories, interpret ambiguous information more pessimistically, and allocate more attention to potential threats than happier people [7]. In animal welfare science, affect-modulated cognition is termed cognitive bias.

Most cognitive bias research on animals has focused on judgement biases (reviewed by [8,9,10,11]). These are usually measured by training animals to respond differently to two unidimensional stimuli, which yield relatively positive- and negative-valence outcomes. For experimental trials, ambiguous intermediate “probe” stimuli are introduced. Responding to probes as though to the positive stimulus is interpreted as an optimistic judgement bias characteristic of positive-valence states; responding as though to the negative stimulus is interpreted as a pessimistic judgement bias indicative of negative-valence states. For instance, Harding et al. (2004) [12] trained rats (*Rattus norvegicus*) to press a lever after one tone for food but not to press it after a different tone to avoid a burst of white noise. When they played an intermediate tone, rats housed in stressful conditions pressed the lever in significantly fewer trials than non-stressed individuals, suggesting a stronger inclination to expect the unpleasant outcome. Variants of the judgement bias task (JBT) have since been developed for other mammals (e.g., rhesus macaques [13], *Macaca mulatta*, sheep [14], *Ovis aries*, dogs [15], *Canis lupus familiaris*, and bottlenose dolphins [16], *Tursiops truncatus*), birds (e.g., chickens [17], *Gallus gallus domesticus*, and starlings [18], *Sturnus vulgaris*), and insects (e.g., fruit flies [19], *Drosophila melanogaster*, and honeybees [20], *Apis mellifera*).

However, JBTs have limitations. Lengthy training periods are time-consuming for researchers, impractical in applied settings, and lead to attrition of subjects. The effect of stress on learning [21,22,23] may also cause a selection bias, with animals in negative-valence states less likely to meet the inclusion criteria [10]. Furthermore, subjects tested repeatedly can learn the probes are unreinforced, making them less likely to respond [24]. Finally, JBTs require well-designed controls for non-valence confounds, such as arousal, motivation, distraction, and general activity (see References [9,10] for further discussion). Cognitive bias paradigms that require less training and fewer controls would help to resolve these issues.

Another class of cognitive bias, attention bias, describes the differential allocation of attentional resources towards one stimulus compared to others. Unlike JBTs, measuring attention biases often requires little or no training and does not depend on interpreting optimistic and pessimistic responses. More fundamentally, external stimuli determine internal valence via attentional processes. In humans, attention biases prioritizing negative information are implicated in the onset and persistence of affective disorders, such as anxiety and social phobia [25]. The stimuli animals attend to also underpin their experience of the world and, ultimately, their wellbeing. Therefore, attention biases warrant investigation [9,26,27].

Attention researchers distinguish between different aspects of attention: initial engagement (attentional capture or orienting, which is enhanced for threat-relevant stimuli [28]), maintenance of attention towards a stimulus (permitting detailed processing), and disengagement (which facilitates shifting to other stimuli) [29]. Human clinical studies have found that affect impacts attention at each stage, from faster engagement to enhanced maintenance and facilitated [30,31] or impaired [32] disengagement. Overall, the evidence shows that clinically anxious people look towards threatening information faster and for longer than non-anxious people [33].

In animal welfare science, attention modulated by the observer’s affective state is typically referred to as “attention bias”. However, the human literature also recognizes attention biases unrelated to affect. For example, people locate inverted letters amongst upright letters faster than upright letters amongst inverted letters [34]. This attention bias towards novelty is not induced by either stimulus valence or the observer’s affective state. To maintain clarity, then, we suggest welfare scientists adopt the term affect-driven attention biases (ADAB). This is measured using attention bias tasks (ABTs; reviewed by [35]). Developed for human research and adapted for animals, ABTs quantify attention allocation to experimental presentations of stimuli. Gaze might be tracked directly or reaction-times recorded to specific cues.

In this review, we evaluate ADAB as a welfare indicator and summarize studies conducted with animals to date. We first outline a nomenclature based on common terminology in human psychology, which will facilitate integration between the literatures (see Table 1). Next, we review studies exploring ADABs in a welfare assessment context and introduce the most widely used ABTs, focusing on their potential as welfare indicators. Finally, we suggest future directions for ADAB research. In this paper, “attention” refers to overt visual attention, typically based on eye gaze. We aim to comprehensively review studies using overt visual attention as the response variable in ADAB tasks, while acknowledging that attentional resources may be attributed to information acquired through other sensory modalities.

## 2. Literature Review: Methods

We aimed to exhaustively review the ADAB literature to date. In March 2018, the Web of Science database was searched with the term “attention bias animal welfare” (26 results). This was updated at the end of July 2018. References to “attention” or “attention bias” were also identified in previous reviews of cognitive bias [7,8,9,10,11] and ABTs for animals [36,37]. In addition, the references in papers identified through these methods and the papers citing them were systematically searched, as well as papers citing the reviews. Titles and abstracts were read to ascertain the relevance of search results. Selection criteria were that subjects must have been tested in different valence conditions, and their attention measured towards an emotional stimulus or stimuli. Animal welfare researchwhere the authors described their findings as an attention bias was also included. Table 2 summarises studies that met these criteria.

## 3. Literature Review: Results and Discussion

We identified 12 ADAB studies, which investigated eight species and used five ABT methodologies. These involved negative-/positive-valence treatments (five studies), negative/control treatments (five studies), inferences of valence from behaviour (one study), and testing animals with different affect-related personality profiles (one study). Of the 10 studies that manipulated affective state, two compared long-term differences in housing, three used pharmacological treatments, and five implemented non-pharmacological manipulations in the week before testing. Stimulus presentations were either negative-valence (one study), negative/neutral (three studies), negative/positive (five studies), negative/neutral/positive (one study), positive (one study) or positive/neutral (one study). All but one study identified significant treatment differences potentially attributable to ADABs. We now discuss this body of research in the context of ABTs from cognitive psychology and other attention bias studies on animals. In particular, we focus on state ADABs, rather than trait affect, and experiments where attention biases were not confounded with judgement biases, which have been reviewed elsewhere [7,8,9,10,11].

### 3.1. Looking Time Tasks

The simplest ABTs are looking time tasks (reviewed by [37]). Originally developed for preverbal human infants [48], they directly measure subjects’ gaze patterns towards visual stimuli, which can be presented either singly or simultaneously. Single-presentation tasks compare looking times between successive trials and reveal which aspects of a stimulus are attended or avoided in the absence of distractions. By contrast, dual-presentation methodologies (also known as the preferential looking paradigm or visual paired comparison) introduce competition between stimuli for processing [49].

Although the preferential looking paradigm had previously been used to address questions related to social attention (e.g., [50]), the first study measuring ADAB was conducted by Bethell et al. (2012) [26] with rhesus macaques. Monkeys were shown two images of conspecifics simultaneously (one with an aggressive expression, the other with a neutral expression) and attention bias was quantified as more time spent looking at one image than the other. Monkeys were tested after a negative-valence manipulation (veterinary health-check) and during a positive-valence manipulation (enhanced enrichment). The macaques showed an attention bias towards the aggressive expression: they looked towards the aggressive face faster than the neutral face. However, maintenance of attention towards the aggressive face was mediated by the affect manipulation. Monkeys continued to look at the aggressive expression during enrichment but were faster to look away following the vet check (and continued to avoid the face for the rest of the trial).

Another ADAB looking time paradigm has recently been validated for sheep [41,42] and cattle [43] (*Bos taurus*; see also Reference [51]). Subjects were moved to a test arena with food available and a hatch was opened for 10s to reveal a dog (a threatening predator stimulus). The response variables were looking time towards the dog and towards the closed hatch after the dog’s removal, as well as latency to feed. In both sheep and cattle, looking time towards the hatch increased with the administration of anxiogenic drugs and decreased with anxiolytics.

Two further studies have investigated ADAB in sheep (see also Reference [52]). Verbeek et al. (2014) [39] demonstrated that food-deprived sheep spent more time interacting with an empty bucket associated with food, which the authors interpreted as enhanced attention. In a longer-term study, Vögeli et al. (2015) [40] kept flocks in two housing conditions, either enriched and predictable to cause positive-valence mood states or unenriched and unpredictable to induce negative-valence mood states. Subjects were then shown videos of other sheep engaged in aggressive, affiliative, and non-social behaviours. Both treatments spent the most time oriented towards the aggressive behaviours and the least towards the affiliative behaviours. Negative-valence sheep spent longer oriented towards the stimuli overall, however, which may have been an ADAB to social information. A video-based preferential looking paradigm was also recently developed for sheep [53], although this study did not include an affect manipulation. Unlike in Vögeli et al., no significant differences were found between the positive (conspecifics) and negative stimuli (dogs).

An additional looking time experiment explored ADAB in starlings. Brilot et al. (2009) [38] switched off the cage lights, added food, and exposed the birds to either a negative-valence treatment (alarm calls, predator calls, and white noise) or a control treatment (conspecific calls). When the lights came back on, stimuli mimicking predator eyespots also appeared and competed with the food for attention. However, there were no treatment differences in time spent orienting towards the stimuli. Brilot et al. attributed this to eyespots not being inherently aversive.

As measures of ADAB, looking time tasks have many benefits. Quantifying eye gaze directly avoids potentially confounding proxies and allows different aspects of attention to be distinguished. Moreover, gaze patterns across stimuli can be observed. For instance, Somppi et al. (2016) [54] found that dogs fixate on certain facial features, such as the eyes, and this effect was influenced by the valence of the facial expression. Measuring untrained looking behaviour is also useful when conditioning would be impractical or impossible, such as research with wild animals and marine species. By suspending objects from a ship, for example, Siniscalchi et al. (2012) [55] measured the looking time of striped dolphins (*Stenella coeruleoalba*) in the Mediterranean Sea. Other paradigms have been successful with free-ranging macaques [56,57,58,59,60]. Future research might also investigate neglected taxa, like recent studies measuring looking behaviour towards conspecific [61] and predator [62] stimuli in lizards.

However, as an indicator of valence, looking time can be difficult to interpret. In Bethell et al.’s rhesus macaque study [26], the authors predicted that stressed monkeys would allocate more attention towards the threatening faces, but looking times were actually shorter in this treatment. Human studies have also associated negative-valence states with both attention to threat (e.g., [34]) and threat-avoidance (for a review, see Reference [63]). While this directionality issue is problematic for attempts to measure valence, it may be overcome by varying stimulus intensity. Human research has identified avoidance of low-level threat and attention to high-level threat in nonclinical populations [64,65]. Demonstrating this effect in macaques may require more objective methods for classifying stimuli. For example, subjective experimenter ratings could be replaced by the Macaque Facial Action Coding System (MaqFACS [66]).

Furthermore, some ADAB studies have used crude measures of looking time, such as how often and for how long the subject’s head is up (e.g., [38]). More precise methods involve manually coding eye gaze from video footage (e.g., [26,67,68]) and automated eye-tracking (see References [37,69,70]; human studies reviewed by References [71,72,73,74]). The latter is fast, objective, and accurate, but also expensive and needs modifying for new species (e.g., marmosets [75], *Callithrix jacchus*, peafowl [76], *Pavo cristatus*, and archerfish [77], *Toxotes chatareus*). Although impractical outside controlled conditions, eye-trackers have been successfully mounted to freely-moving ring-tailed lemurs [78,79] (*Lemur catta*). While existing tasks typically measure attention to static images, responses to photographs are often quantitatively weaker or qualitatively different than responses to moving images or the objects themselves [80,81]. Researchers could, therefore, experiment with videos [82,83,84,85,86], computer animations [87,88], models [89], and real animals or objects [41,42,43,54].

Collectively, these studies demonstrate the potential of looking time tasks to investigate ADAB, with research on macaques, sheep, and cattle suggesting it is affect-modulated. Similar paradigms would be suitable for any animal with measurable eye gaze, including birds and reptiles, and the simplest methods do not require training. Looking time tasks could, therefore, be adapted to a range of species and situations.

### 3.2. Emotional Stroop Tasks

The emotional Stroop task [90] (reviewed by Reference [91]) measures how much an individual is distracted by emotional information while they perform an otherwise neutral cognitive task. Typically, participants are instructed to name, as quickly and accurately as possible, the colour in which words appear on a screen. Anxious populations are slower to name the colour of negative-valence words (e.g., pain) than neutral words (e.g., gain; see Reference [92] for a review), an effect not seen in non-anxious people. Variants of the task using facial expressions (neutral, aggressive, and happy) instead of words produce similar results [93]. The emotional Stroop effect is interpreted as negative affect enhancing attentional capture by negative distractor content [94,95,96], although the task does not distinguish capture, maintenance or disengagement of attention. It also does not rule out alternative explanations, such as freeze response [97].

An emotional Stroop task was recently developed for chimpanzees [44] (*Pan troglodytes*), which were trained to press a blue-framed square on a touchscreen, but not a yellow-framed square. The chimpanzees’ response-times were then recorded to blue-framed squares containing images of either the veterinarian or non-threatening humans. Subjects were slower to touch the blue frame when it contained a picture of the vet, a slowing effect that was stronger when they had recently undergone a veterinary procedure. The authors attributed this result to ADABs; specifically, attentional capture by stimuli associated with negative-valence states (see also Reference [98]).

However, this paradigm required extensive training. Of 16 chimpanzees conditioned on the blue-frame/yellow-frame discrimination task, only seven reached the study’s inclusion criteria. Even those needed 900–6700 trials [98]. This extended training period and attrition of subjects suggests the paradigm may be impractical for welfare assessment. More fundamentally, reaction-times in the emotional Stroop paradigm can reflect motor action biases rather than attention biases, so changes in response are difficult to interpret in terms of attention. This is less of an issue in other ABTs, which measure reaction-times to valence-neutral targets after the emotional stimuli have disappeared.

Other studies have quantified emotional Stroop effects by observing how emotional stimuli distract from an attention-demanding task. Performance decrements were observed in amazon parrots (*Amazona amazonica*) exposed to humans during a foraging task, and this effect correlated with subjective ratings of the birds’ neuroticism [45]. Landman et al. (2014) [99] found that threatening facial expressions distracted macaques from a visual task, whereas Bellegarde et al. (2017) [100] reported that sheep learnt a discrimination task faster when it involved negative-valence facial stimuli than valence-neutral images.

Stroop effects have also been studied in the context of learned helplessness. A classic model of depression, learned helplessness describes the unresponsiveness of animals that cannot escape repeated uncontrollable stressors, typically electric shocks (for a review, see Reference [101]). One consequence is an attention bias towards goal-irrelevant external stimuli [102]. In studies on rats, for example, the performance of subjects in learned helplessness treatments was equivalent to controls on an automated cognitive task, but they were slower and made more errors when the experimenter was present as a distraction [103,104]. Eliminating the trained task completely, Rodd et al. (1997) [105] investigated whether learned helplessness-induced attentional shifts influenced an innate behaviour in chickens. Duration of tonic immobility, a catatonic response to restraint, increases with perceived threat. In the presence of eyespots, chickens in a learned helplessness condition were motionless for longer than control birds, but they recovered faster in the presence of unaffected conspecifics. Despite indicating a putatively negative-valence state, we do not consider these examples of ADAB because they are attention biases towards external stimuli rather than emotional stimuli per se [102]. Learned helplessness research nonetheless demonstrates that attentional shifts are associated with depression-like states as well as anxiety, and similar experiments could investigate ADAB.

The conventional emotional Stroop task has only been tested with chimpanzees, although emotional Stroop effects are observed in various species. Given the time requirement of training and issues in interpretation, the human paradigm is unlikely to translate to the applied setting. However, neutral task performance or behavioural shifts in the presence of a threatening stimulus are a simple, adaptable approach to measuring ADAB in animals.

### 3.3. Dot-Probe Tasks

The dot-probe paradigm [106] (reviewed by Reference [36]; metanalyses by References [34,107]) presents participants with two stimuli on a screen. Stimuli may be words (e.g., threatening/neutral pairs [106,108]) or images (e.g., different facial expressions [109,110]). After a fixed duration, both stimuli disappear, and one is replaced by the “dot-probe”—a neutral target subjects must respond to. Faster reaction-times to the target indicate the participant’s attention was already fixed on that location, whereas slower reaction-times suggest their attention shifted from the other location. In humans, dot-probe studies pairing negative and neutral stimuli have found attention to threat is stronger in anxious [34,111] and depressed [112] people, and during high-stress situations [113,114].

To our knowledge, no published studies have tested for ADAB in animals using the dot-probe paradigm. However, Kret et al. (2016) [115] identified attention biases to positive-valence social cues in bonobos (*Pan paniscus*) with a dot-probe task. When presented with image pairs of conspecifics performing emotion-regulatory and neutral behaviours, subjects responded faster if targets replaced the emotional stimuli, but only for certain behaviour classes. Effects were significant for grooming and sex, but not play or distress. Kret et al. explained the attention biases towards affiliative social stimuli as an adaptation to facilitate bonobos’ characteristic conflict-resolution and emotion-regulation strategies [116]. While effect sizes may be smaller [117], ADAB studies could further explore responses to positive stimuli, such as play faces [118,119], gentle handlers [51], and food [39].

Several authors have also trained macaques on dot-probe tasks (e.g., [120,121]). King et al. (2012) [122] observed a baseline attention bias towards threatening stimuli (open-mouth conspecifics), which was unaffected by testosterone administration. In a study with Japanese macaques (*Macaca fuscata*), Koda et al. (2013) [123] used stimulus pairs of new-borns and adults, but did not find a significant difference in reaction-times.

The dot-probe task provides a snapshot of attention allocation, and questions about orienting and maintenance of attention can be answered by adjusting the length of stimulus presentation. For example, work with humans has shown attention bias for threatening stimuli presented below the temporal threshold for awareness. Testing with multiple durations offers a better understanding of the aspects of attention involved, with shorter durations measuring engagement (e.g., [123]) and longer durations measuring disengagement (e.g., [120]).

Future dot-probe studies could investigate whether affect manipulations influence reaction-times and explore the aspects of attention underpinning ADAB. While this task requires relatively little training, touchscreens are best suited to laboratory settings and dexterous subjects, especially primates.

### 3.4. Emotional Spatial Cueing Tasks

Spatial cueing tasks [124] also quantify attention biases through reaction-times to a valence-neutral target. Subjects first fixate on a point in the centre of a screen. Their objective is to respond as quickly as possible to the target, which can appear on either side. Before the target’s arrival, a cue signals its position. This is usually located where the target will be, but is on the other side of the screen in a minority of trials. Reaction-times when the cue correctly predicts the target’s location indicate attentional engagement towards the cue, whereas reaction-times when the target and cue appear in different locations indicate disengagement from the cue [125,126]. Hence, unlike the dot-probe task, spatial cueing automatically distinguishes between different aspects of attention.

The emotional spatial cueing paradigm involves manipulating the cue’s affective content. For example, Fox et al. (2001) [30] tested anxious and non-anxious people on a task with threatening words and faces as cues. There were no treatment differences in engagement to the cues, but a significant difference in disengagement. Anxious individuals were slower to reallocate their attention from the threatening cue to the valence-neutral target.

No studies, to our knowledge, have adapted the emotional spatial cueing task for animals. However, non-valenced predictive cue paradigms have been successful with macaques [127], rats [128], chickens [129], honeybees [130], and archerfish [131]. In the latter study, a touchscreen was suspended above the tank and a symbolic cue predicted the location of a food-delivering target. Fish were trained to hit the targets using mouth-propelled water jets, a natural behaviour for this species. Their reaction-times were faster when the target appeared in the same location as the cue.

To create an emotional spatial cueing task, it would be a small step to introduce affective stimuli into the predictive cue paradigm and test subjects in different valence states. This could be effective with diverse taxa, albeit under controlled conditions.

### 3.5. Visual Search Tasks

In the visual search task, participants are instructed to locate a target stimulus in an array of distractor stimuli (e.g., [28,132]). Faster target detection reveals attention bias for the stimulus, whereas slower detection suggests either the target does not capture attention or does so less than other images in the array.

Marzouki et al. (2014) [46] used abstract shapes with conditioned valence in a visual search task for Guinea baboons (*Papio papio*). Subjects were trained to locate a T-shaped target among seven L-shape distractors. To investigate the influence of affective state, the authors only analysed trials preceded by ostensibly valence-informative behaviours. Reaction-times in trials following negative-valence behaviours were slower than those following valence-neutral or positive-valence behaviours.

However, observational studies inferring affective state from behaviour can be open to interpretation. For example, while Marzouki et al. categorized self-grooming as positive-valence, this displacement activity has been widely linked to stress [133,134]. Resting, analysed as negative-valence, is a biological necessity. It is also a low-arousal activity, suggesting the observed effect could be attributed to affective intensity rather than valence. Well-designed affect-induction experiments can avoid such confounds and demonstrate causality.

Primate response-times have also been recorded towards non-symbolic, emotional images, albeit in studies that did not manipulate subjects’ affect. Chimpanzees located conspecific faces faster than neutral objects [135], and human faces faster when their gaze was directly forward rather than averted [136]. In Japanese macaques, median reaction-times were faster towards target aggressive faces amongst neutral face distractors than vice versa [137] (see also Reference [138]). Macaques also detected snakes quicker than fear-irrelevant stimuli in arrays of neutral distractors [139,140]. Instead of using reaction-times as the response variable, Simpson et al. (2017) [141] measured infant macaques’ gaze across visual search arrays with an eye-tracker. This eliminated training and potential motor response biases.

Other taxa have also been trained on visual search tasks, including recent work with barn owls [142,143] (*Tyto alba*), zebrafish [144] (*Danio rerio*), and bumblebees [145] (*Bombus terrestris*). The latter study conditioned bees to associate specific colours with rewards, which they could detect in arrays of distractor colours. As far as we are aware, no non-primate studies have investigated whether affective state influences performance on visual search tasks. However, research on judgement bias has validated affect manipulations for bees [20,146].

Visual search tasks have recently been criticised in cognitive psychology, because searching is heavily influenced by the intensity of emotional stimuli, as well as their valence [147,148,149]. In a systematic reanalysis of human studies, Lundqvist et al. (2013) [150] concluded that happy facial expressions captured attention faster than angry expressions when they were rated higher on arousal indices, but this effect was reversed when the angry expressions were rated as higher arousal. Another study exposed subjects to either an arousing negative-valence sound, an arousing positive-valence sound or a neutral sound before they completed a visual search task [151]. Both high-arousal sounds had similar effects on performance, regardless of valence. To avoid these issues, visual search experiments should control for the effects of arousal (see Reference [152]).

The complexity of visual search tasks, coupled with the issue of arousal, suggests they are not an immediate priority for ADAB research. However, primate studies could investigate the influence of different affect manipulations on search behaviour.

### 3.6. Related Paradigms

In addition to standard ABTs, other tests and metrics might also measure ADAB. Based on human paradigms [153], the five-choice serial reaction time task [154] (5-CSRTT; reviewed by Reference [155]) presents subjects with five apertures and requires them to approach the one just illuminated. It is framed in terms of measuring attention. In a study on pain in rats, Boyette-Davis et al. (2008) [156] tested subjects injected with formalin on the 5-CSRTT. Formalin-treated rats made fewer approaches, which the authors interpreted as a failure to attend to the task when in pain (see also Reference [157,158]; pain-induced cognitive impairment reviewed by Reference [159]). Behavioural data confirmed that subjects that did not receive morphine were in the most pain, and they showed the highest rates of locomotion in open field tests, suggesting their failure to respond was not due to reduced activity. Additionally, data from trials when they did approach indicated responses were no slower in the pain group.

JBTs may also measure ADABs. Like Harding et al. (2004) [12], Parker et al. (2014) [47] tested rats on tones intermediate between two reference tones associated with differently valenced outcomes, but they also recorded responses to dual presentations of both trained tones. The authors suggested the latter approach was a novel ABT. In addition, results from JBTs using biologically relevant stimuli might be attributed to ADABs. For example, chicks moved slower down an alley containing predator silhouettes and conspecific/predator morphs following social isolation [160], an effect reversed by anxiolytic treatment [161]. This may indicate an ADAB away from threatening stimuli. Other studies have tested ambiguous eyespots on starlings [38] (no effect) and morphed facial expressions on goats [162] (*Capra aegagrus hircus*; no affect manipulation).

However, there is insufficient evidence to infer that attention underpins “judgement” biases. In the dual-presentation task, for instance, responses relied on the rats hearing one or both tones (attention), categorizing the presentation as either positive or negative (judgement), remembering the lever associated with each outcome (memory), and choosing which to press (decision-making). More research is needed to identify which cognitive faculties contribute to observed biases, bearing in mind they may act synergistically [163,164]. Parker et al. did not specify why their dual-presentation JBT should measure attention biases any more than the ambiguous-probe paradigm, but presumably reasoned that valence-conditioned stimuli would influence attention more than unfamiliar ambiguous stimuli.

Paul et al. (2005) [7] suggested some personality tests might also quantify ADAB. For example, novel object and human reactivity tests [165] often record looking time, latency to first touch, and subsequent interactions, which could be measures of attention and potentially affect-modulated. Startle tests [166,167], which expose subjects to sudden acoustic or visual stimuli, also measure looking behaviour towards the source. Stress potentiates the startle reflex in humans [168], and it is associated with negative-valence conditions such as clinical anxiety [169] and chronic pain [170]. Destrez et al. (2013) [171] explored the potential of these paradigms as indicators of ADAB by subjecting both chronically stressed and control lambs to a battery of personality tests. The negative-valence lambs made fewer contacts with a human entering their pen and looked towards a novel object for shorter bouts, although total looking time did not differ and there were no treatment differences in a startle test. Negative-valence states increase the startle response in rodents and macaques, however [172,173].

Finally, vigilance, or scanning the environment for threats, is influenced by fear and anxiety [174]. For example, Brilot et al. (2012) [27] discovered that, following an alarm call, starlings with access to a water bath spent less time vigilant and more time with their heads down compared to those without. The authors attributed this to the importance of bathing in maintaining feather condition, which improved the birds’ flying ability and lowered their vulnerability to predators. In livestock, anxiolytic treatment reduces vigilant behaviour during isolation tests [175] and after exposure to a potential predator [41,42,43]. Vigilance in cows is also lower in the presence of gentle, compared to aversive, stock people [51]. Furthermore, wild animals display heightened vigilance under dangerous or stressful conditions (reviewed by [176]). In playback experiments, coots (*Fulica atra*) spent longer scanning their surroundings after dog barks than control sounds [177]. Observational studies have also associated vigilance with predation risk in African ungulates [178], human disturbance in Japanese cranes [179] (*Grus japonensis*), and proximity to neighbouring territories in spider monkeys [180] (*Ateles geoffroyi*). Although these findings do not meet our stimulus-directed definition of ADAB, they demonstrate how attention biases might be measured in the field and provide an insight into their adaptive function.

## 4. Outstanding Questions and Future Directions

### 4.1. Other Sensory Modalities

Although we have focused on visual attention, other sensory modalities warrant investigation (see Reference [181]). This is illustrated by judgement bias studies, which have used auditory [12], olfactory [20], gustatory [182], and haptic [183] cues. Previous ABTs have also experimented with acoustic stimuli, such as starling alarm calls [38] and threatening dog barks [184], but most measured looking time towards the source. For some species, however, other response variables might be more conspicuous and accurate. Ruminants have a wide field of vision, for instance, rendering head orientation a potentially unreliable proxy for eye gaze. Instead, ear posture and movement signals affective state in several livestock species (e.g., cattle [185,186], sheep [53,187,188,189], goats [190], and pigs [191], *Sus scrofa domesticus*), as well as dogs [192] and mice [193] (*Mus spicilegus*). A preferential hearing paradigm might replace the competing images with a positive-valence conspecific vocalization and a negative-valence predator vocalization. Ear position would indicate ADAB. Like eye-trackers, an automated ear tracking system has even been developed for sheep [187]. However, ear postures can be purely communicative and indicate arousal as well as valence [185].

Non-visual ABTs would also be more biologically relevant for animals where vision is not the primary sense. Olfaction in particular is often overlooked by animal welfare scientists [194], despite being a dominant sensory modality for species widely used by humans, such as dogs [195], rats [196], and chickens [197]. Moreover, it is integral for communication and information-gathering in arthropods [198]. Cárdenas et al. (2012) [199] presented predatory spiders (*Zodarion rubidum*) with a control chamber and an experimental chamber, which airborne odours from different prey species were channelled into. Approaches into the experimental chamber therefore indicated attractive kairomones. While olfactory stimuli are inherently difficult to work with [194], ADAB researchers might use this method to investigate whether affect influences responses to food, conspecific, and predator odours. Such non-visual ABTs may facilitate research on commercially important and poorly studied taxa.

### 4.2. Effect Specificity

In humans, attention biases are often stimulus- or motivation-specific [117]. Combat veterans with post-traumatic stress disorder exhibit facilitated engagement and impaired disengagement when viewing war-related stimuli, but not disgust, neutral or positive-valence stimuli [200]. In emotional Stroop tasks, anxiety sufferers concerned about physical threats are slower to name the colour of words like “attack” and “illness”, whereas those with social worries are distracted by “incompetent” and “stupid” [64,65]. Insomniacs struggle to disengage from sleep-related stimuli [201]; addicts are biased towards opiates [202], cigarettes [203], and alcohol [204]; and, in healthy populations, food cues are more salient to hungry people [205,206,207]. These findings suggest blanket valence-based interpretations of attention biases may be inappropriate in animal welfare science, as well. Whereas JBT results can be understood as a correlation between optimism and valence, ABTs defy simple, overarching explanations.

Nonetheless, the studies reviewed herein demonstrate that ADABs can reveal specific emotions, motivations, aversions, and preferences. In the aforementioned sheep and cattle ABT, for example, anxiogenic and anxiolytic drugs modulated attention allocation towards a dog [41,42,43]. This threat-based task did not measure negative valence generally, but fear and anxiety specifically. Verbeek et al. (2014) [39] found that food-motivated sheep attended to a food-delivering bucket, while the emotional Stroop task for chimpanzees [44] used a contextual aversive stimulus (images of the veterinarian) that potentiated ADABs when subjects had recently encountered the vet.

Dawkins (2003) [208] defined animal welfare without recourse to affect, arguing it could be distilled into two questions: Is the animal physically healthy and does the animal have what it wants? Although other variables can increase attention towards a stimulus [41], ABTs might be a “quick-and-dirty” method to identify promising avenues for labour-intensive preference, motivation or aversion tests (reviewed by [209]). Hence, ADABs might help to answer Dawkins’ second question: Do animals have what they want?

### 4.3. Attentional Scope

Whereas ADABs towards specific stimuli might not indicate general valence, attentional scope may do so. The broaden-and-build theory of positive emotions [210,211] (reviewed by Reference [212]) proposes that, since positive-valence states often reflect overall wellbeing, contented individuals can devote resources to exploration, learning, and building up resources. Positive affects are, therefore, associated with a broad attentional scope and more attention allocated towards the periphery of the visual field (i.e., seeing the forest rather than the trees). On the other hand, negative-valence states are typically directed towards specific threats, so they narrow attentional scope and maximize attention towards the centre of the visual field [213] (i.e., seeing the trees rather than the forest). This has been demonstrated using various attentional tasks. In the Kimchi test [214], for instance, a target shape is presented, followed by two comparison shapes. Subjects are instructed to select the comparison most like the target. To test broaden-and-build theory, Gasper and Clore (2002) [215] showed participants a larger shape consisting of different smaller shapes as the target. This was either a triangle consisting of three squares or a square consisting of four triangles. After a positive-valence affect manipulation, subjects chose the larger shape as a closer match than the component shape more than subjects in a negative-valence condition, suggesting a broader attentional scope. Similar experiments might also indicate positive emotions and general wellbeing in animals (although, see Reference [216]).

### 4.4. Attention Bias Modification

Animal welfare scientists study attention biases as a symptom of negative-valence states, but some cognitive models of affective disorders identify them as a proximate cause (e.g., References [217,218,219]; reviewed by [220]). For instance, exaggerated attention to threat in anxious populations generates a positive feedback loop that reinforces existing biases [221]. A controversial new treatment aims to disrupt this harmful bidirectional relationship: attention bias modification (reviews and metaanalyses by References [25,222,223,224,225,226,227]). Using simple computer-based tasks, attention bias modification repeatedly presents subjects with valence/neutral stimulus pairs, such as angry and neutral facial expressions. For this example, correct responses would always require focusing on the neutral stimulus, training participants to divert attention away from threats through operant conditioning. In a modified dot-probe task, for instance, probes only ever appear behind neutral stimuli (e.g., Reference [228]). Although several recent studies have reported null effects (e.g., References [229,230,231,232]), the more promising tasks could be adapted for animals (e.g., the positive search paradigm [233,234]). For anxious individuals or situations where chronic stress is unavoidable, such as some primate laboratories, attention bias modification might be a cost-effective way to enhance mood states. However, this is not a substitute for good housing and husbandry.

## 5. Conclusions

We reviewed the literature on affect-driven attention bias as a welfare indicator and identified 12 studies to date. The initial results are promising. In chimpanzees, macaques, sheep, and cattle, affect manipulations have modulated attention towards or away from emotional stimuli, as well as the speed and duration of fixation. Both positive- and negative-valence states have been studied, although most animal research has concentrated on fear, anxiety, and threat biases. While welfare scientists were quick to recognise the potential of judgement biases, however, research on affect-driven attention biases remains comparatively limited. Nonetheless, methods might be developed for a range of taxa, including birds, reptiles, fish, and insects, and tested in both captive and free-range settings. Different attentional tasks measure different aspects of attention, but the looking time, dot-probe, and spatial cueing paradigms are especially promising. Future studies could use them to distinguish engagement and disengagement of attention, investigate effect specificity, and explore attentional scope as a welfare indicator. Attention bias modification might also ameliorate negative-valence moods in chronically stressed animals. By describing potential methodologies and evaluating the existing literature, we hope this review stimulates such research in diverse settings and species.

## Figures and Tables

**Table 1:** Glossary.

Term	Definition
Affect	Umbrella term for all valenced experiences, including moods, emotions, and feelings.
Affect-driven attention bias (ADAB)	An attention bias towards or away from emotional information that is influenced by the observer’s affective state. Often labelled “attention bias” in the animal welfare literature.
Affective state	A temporary affect, e.g., emotions or moods.
Attention	The selective allocation of cognitive resources to particular information.
Attention bias	The preferential allocation of attentional resources towards one form of information over another.
Attention bias task (ABT)	An experimental paradigm that presents subjects with stimuli and records how their attention is allocated. Examples covered here include looking time, emotional Stroop, dot-probe, emotional spatial cueing, and visual search tasks.
Attention to emotion	Attention allocated towards emotional stimuli.
Attention to threat	Attention allocated towards threatening stimuli.
Avoidance of threat	Attention allocated away from threatening stimuli.
Cognition	The mechanisms, such as attention and judgement, that animals use to gather, process, and store information.
Cognitive bias	In the animal welfare literature, an umbrella term for cognitive processes influenced by affect, e.g., attention and judgement biases.
Disengagement (of attention)	The allocation of attention away from a stimulus previously attended to.
Emotion	Stimulus-directed affective state. Consists of behavioural, physiological, and cognitive components, and may occur outside awareness (cf. “Feeling”).
Engagement (of attention)	The initial allocation of attention towards a stimulus. Limited attentional resources mean engagement to one stimulus may draw resources away from other tasks.
Feeling	Subjective, experiential element of affect. Because animals’ feelings cannot be reported directly, we rely on indirect indicators that can be objectively measured, e.g., behaviour, physiology, and cognitive biases.
Judgement Bias	A cognitive bias where affect influences judgements about the affective value of ambiguous stimuli. Positive affect is associated with optimistic judgements; negative affect is associated with pessimistic judgements.
Judgement bias task (JBT)	A task that uses judgements of ambiguous stimuli as an indicator of affect. Typically, subjects are trained to react differently to two stimuli to achieve relatively positive- and negative-valence outcomes. Responses to subsequent presentations of intermediate “probe” stimuli indicate whether subjects judge them more positively (optimistic responses) or negatively (pessimistic responses).
Mood	A long-lasting affective state that reflects the cumulative impact of emotion over preceding days, weeks or months.
Motivation	Drives arising from internal signals that compel behaviour to meet basic biological needs, e.g., hunger and thirst.
Overt attention	A measurable proxy for attention, such as movements of the eye with respect to stimuli.
Personality	Behavioural and psychological traits with inter-individual variation but intra-individual consistency across time and contexts.
Trait affect	Affect stable within individuals over time. A personality trait that does not encompass transient emotions or moods.
Vigilance	Scanning the environment for potential threats (may occur in the absence of threatening stimuli).

**Table 2:** Affect-driven attention bias studies on animals.

Species	Ref.	*N*	Stimuli	Measure/Manipulation of Affect	Measure of Attention	Findings
Starling	[38]	32	Eyespots, ambiguous eyespots, ctrls	NV: predator call, alarm call, white noise	Orienting towards stimuli	No effect
Sheep	[39]	41	Empty food bucket	NV: food-deprivation	Detection/approach latency, object interaction	No effect for detection/approach latency; NV sheep interacted longer
	[40]	29	Aggressive, affiliative, and non-social behaviours (video)	NV: unpredictable, unenriched housing; PV: predictable, enriched housing	Orienting towards stimuli	Time oriented towards stimuli (all subjects): aggressive > neutral > affiliative. NV: oriented towards stimuli longer overall
Dual-Presentation Looking Time Task						
Rhesus macaque	[26]	7	Aggressive/neutral faces	NV: post-vet health-check; PV: enrichment	Eye gaze	All monkeys initially oriented faster towards aggressive faces; NV monkeys disengaged faster from aggressive faces
Starling	[27]	14	Alarm call/food	NV: no water bath	Head up/down duration	NV birds longer head-up bout and shorter head-down bout duration
Sheep	[41]	60	Dog/food	NV: anxiogenic; PV: anxiolytic	Looking time, head up duration, latency to eat	Looking duration/head up/latency to eat: NV > ctrl > PV
	[42]	60	Dog/food	NV: anxiogenic; PV: anxiolytic	Looking time, head up duration, latency to eat	Looking duration/head up/latency to eat: NV > ctrl > PV
Cattle	[43]	36	Dog/food	NV: anxiogenic; PV: anxiolytic	Looking time, head up duration, latency to eat	NV looking duration/head up/latency to feed > ctrl; no effect for PV
Emotional Stroop Task						
Chimpanzee	[44]	7	Vet (negative-valence) and other humans	NV: post-vet health-check	Colour discrimination task RTs	All subjects: RTs slower to touch correct colour when it contained image of the vet than non-threatening humans. NV subjects: slower than ctrls to touch the correct colour when it contained image of the vet
Orange-winged amazon	[45]	20	Human	Subjective personality assessment	Spatial memory task RTs	Negative correlation between neuroticisim ratings and task performance (suggests greater distraction from human present)
Visual Search Task						
Guinea baboon	[46]	6	T-/L-shapes (conditioned valence)	NV and PV behaviours (observational)	RT to the target	RT: NV > ctrl > PV
Dual-Presentation Judgement Bias Task						
Brown rat	[47]	16	Tones (conditioned valence)	NV: unpredictable housing	Lever pressed (binary) and RT to lever press	Ambiguous-probe and dual-presentation JBT congruent: NV rats pressed positive lever (optimistic responses) more than ctrls. Suggests AB towards negative-valence stimulus

Abbreviations: Negative valence (NV), positive valence (PV), reaction-time (RT), attention bias (AB), control (ctrl).
